# Impact of Growth Conditions on the Viability of *Trichoderma asperellum* during Storage

**DOI:** 10.3390/microorganisms11041084

**Published:** 2023-04-21

**Authors:** Alina Rimkus, Agne Namina, Marija Tereze Dzierkale, Oskars Grigs, Maris Senkovs, Simona Larsson

**Affiliations:** 1 Bioefekts Ltd., 30 Livzemes Street, LV-2169 Salaspils, Latvia; rimkus.alinaa@gmail.com (A.R.); agne.namina@gmail.com (A.N.); info@bioefekts.lv (M.T.D.); maris.senkovs@lu.lv (M.S.); 2 Laboratory of Bioengineering, Latvian State Institute of Wood Chemistry, Dzerbenes Street 27, LV-1006 Riga, Latvia; oskars.grigs@kki.lv; 3 Microbial Strain Collection of Latvia, Faculty of Biology, University of Latvia, 1 Jelgavas Street, LV-1004 Riga, Latvia

**Keywords:** *Trichoderma asperellum*, viability, cultivation media, shelf-life, peat

## Abstract

As excellent biocontrol agents and plant growth promoters, *Trichoderma* species are agriculturally important. *Trichoderma* spp. cultures can be produced using solid-state or submerged cultivation, the latter being much less labor intensive and easier to control and automate. The aim of the study was to investigate the ability to increase the shelf-life of *T. asperellum* cells by optimizing cultivation media and upscaling the submerged cultivation process. Four different cultivation media were used with or without the addition of Tween 80 and stored with or without incorporation into peat, and viability, expressed as CFU/g, was assessed during one year of storage in an industrial warehouse. The addition of Tween 80 had a positive effect on the biomass yield. The culture medium played a major role in the ability of the mycelium to produce spores, which in turn influenced the amount of CFU. This effect was less pronounced when the biomass was mixed with peat prior to storage. A procedure that increases the number of CFU in a peat-based product formulation is recommended, namely, incubation of the mixture at 30 °C for 10 days prior to storage at 15 °C over an extended period of time.

## 1. Introduction

*Trichoderma* species are ubiquitous in soil and have long been known as excellent biocontrol agents [1] and plant growth promoters [2].

*Trichoderma* can be produced in two ways: through submerged (liquid) fermentation and through solid-state fermentation [3]. Each method has its advantages and disadvantages [4]. However, submerged processes are much less labor intensive and easier to control and automate. In submerged processes, *Trichoderma* is usually cultured in shaken flasks or in industrial-scale stirred bioreactors [5]. Fungal conidia are formed on aerial mycelia and are not usually produced in liquid media [6].

Asexual conidia or spores produced by submerged cultivation are the primary means of protection and preservation of fungal genomes that are beneficial for mycopesticide development [7]. Cultivation parameters (nutrient composition, duration of cultivation, pH, etc.) affect spore and biomass quality, affecting the viability and shelf life [8]. Volatile and non-volatile antibiotic production by *Trichoderma* spp., which renders the fungus antagonistic to a range of pathogenic fungi, was recently investigated in a liquid culture [9].

Many factors, such as the medium and inoculum type [10], method of drying, and the addition of protectants [11] and environmental conditions during storage [12], affect the viability of the formulation derived from liquid fermentation.

Czapek Dox medium or its modified composition is available in submerged cultivation examples of *Trichoderma* spp. [13,14,15], in which varied sucrose concentrations (10–30 g/L), a sucrose source (e.g., molasses), or the addition of extra yeast extract/peptone (1–5 g/L) was used. *Trichoderma* spp. biomass dry weight concentrations of 2–15 g/L were achieved in 4–7-day cultivation processes [13,15]. Malt extract is a known component that is used in microbial and fungus cultivations; however, its use in submerged cultivations of *Trichoderma* spp. has rarely been reported so far. The addition of surfactants is an example of an intervention that can be made during fermentation to extend the shelf life of formulations. The addition of Tween surfactants to the fermentation medium has been reported to have a beneficial effect on fungus mycelial biomass growth [16,17], protein secretion [18], and exopolysaccharide production [19,20] in submerged cultivation processes. Lucatero and co-authors [16] investigated the increase in *Trichoderma* spp. biomass in proportion to the added Tween concentration.

The ideal properties of a soil conditioner containing microorganisms include: (i) a production process that is economical, cost effective, and preferably takes place through submerged liquid fermentation; (ii) preservation against microbial contamination in dry form and low water availability; and (iii) a long shelf-life biomass [21]. A biocontrol preparation may not possess all these properties, but it should have as many of these desirable properties as possible [22]. Different carrier materials have been described in the literature, e.g., composted manure [23], bagasse [24], wood [25], biochar [26], polymeric formulations [27], talc [28], and peat [29], the latter being a frequently used formulation of *Trichoderma* spp. products [30,31], as peat is a widely used substrate for horticulture [32]. Such preparations have been reported to have a shelf life of 3–4 months [25,33]. In some cases, the microorganism was able to achieve viability and a high CFU/g output (up to 8 × 10^6^ CFU/g) when samples were stored for 24 weeks [26,34]. However, commercial peat-based preparations would require a shelf life of at least one year.

The aim of this study was to evaluate the effect of different culture media on *Trichoderma asperellum* MSCL 309 biomass production and the shelf life of fungal spore- and peat-based preparations. *Trichoderma asperellum* strain MSCL 309 was chosen for the viability assessment in the present study, firstly because it was previously isolated from a temperate climate region (Latvia) and taxonomically identified by amplification of the rRNA ITS region with specific primers. *T. asperellum* strain MSCL 309 inhibits the growth of the conifer root and butt rot fungi *Heterobasidion annosum* s.s. and *H. parviporum*, which gives it a high potential for use against phytopathogens. [35] *T. asperellum* MSCL 309 can maintain its viability and antifungal activity for a long time, with or without different post-treatment methods [36]. To investigate the storage conditions, regular and accelerated storage tests [37] were applied. The regular shelf-life storage was applied to mimic the conditions of warehousing that distributors and retail chains might employ.

## 2. Materials and Methods

### 2.1. Maintenance of the Culture and Media Used

*Trichoderma asperellum* MSCL 309 isolate [35] was chosen from the Microbial Strain Collection of Latvia. The fungi were cultivated on malt extract agar (Biolife Italiana, Monza, Italy) plates for 7 days at room temperature, and then the plates were stored at −70 °C for further use.

*Trichoderma asperellum* was cultivated on four different media, both without (M1, M2, M3, M4) and with (MT1, MT2, MT3, MT4) the addition of 0.2% Tween 80 (Sigma-Aldrich, Saint Louis, MO, USA) in a bioreactor and 1% Tween 80 in flasks, with three replicates of each. The media compositions were as follows: malt extract broth (M1) (Bioefekts, Salaspils, Latvia) at 70 mL/L; yeast extract medium (M2) consisted of yeast extract (Biolife, Italy) at 3 g/L, sucrose (Sigma-Aldrich, Saint Louis, MO, USA) at 20 g/L; salt medium (M3)—sucrose at 10 g/L, KH_2_PO_4_ at 1 g/L, MgSO_4_ at 0.5 g/L, FeSO_4_ (Sigma-Aldrich, Saint Louis, MO, USA) at 0.02 g/L, NaNO_3_ (Sigma-Aldrich, Saint Louis, MO, USA) at 2 g/L, and peptone bacteriological (Biolife Italiana, Monza, Italy) at 1.12 g/L; and sugar beet molasses medium (M4)—molasses (KGM, Ropaži, Latvia) at 10 g/L, KH_2_PO_4_ (Lach-Ner, Neratovice, Czechia) at 2 g/L and MgSO_4_ (Chempur, Piekary Śląskie Poland) at 0.2 g/L. The media were sterilized for 20 min for flask cultivations and 30 min for bioreactor cultivations at a temperature of 121 °C and a pressure of 1 atmosphere.

### 2.2. Trichoderma asperellum Cultivation in Shake Flasks

*Trichoderma asperellum* biomass was cultivated in a 2 L flask with a 1 L working volume, in which 1 mL inoculum (*T. asperellum* spores in water suspension) with OD = 0.16 at 540 nm (corresponding to 6 × 10^6^ CFU/mL) was inoculated. The flasks (with three repetitions) were incubated at 25 °C with shaking at 150 rpm for 72 h.

### 2.3. Trichoderma asperellum Cultivation in a Bioreactor

*Trichoderma asperellum* biomass was cultivated in a 15 L stirred-tank bioreactor (EDF-15.1, Biotehniskais centrs, Riga, Latvia) with one standard Rushton turbine (bottom location) and two propeller-type turbines (one located in the middle for up-flow and one above for down-flow) for 65 h at 28 °C. pO_2_ was set at 30 ± 5%, aeration at 1.67 slpm, and stirring at 200–750 rpm. At the end of the fermentation, the pH of the culture liquid was brought to pH 4 with 1 M HCl solution.

Inoculum was prepared from *T. asperellum* statically grown in malt extract broth in flasks for 10 days at 28 °C and then shaken at 120 rpm for 6 h. *Trichoderma asperellum* 5% inoculum with a spore count of 6 × 10^6^–8 × 10^6^ CFU/mL was used. The spore count was determined using a Fuchs–Rosenthal cell-counting chamber.

### 2.4. Storage of T. asperellum in Peat

**Figure 1:**
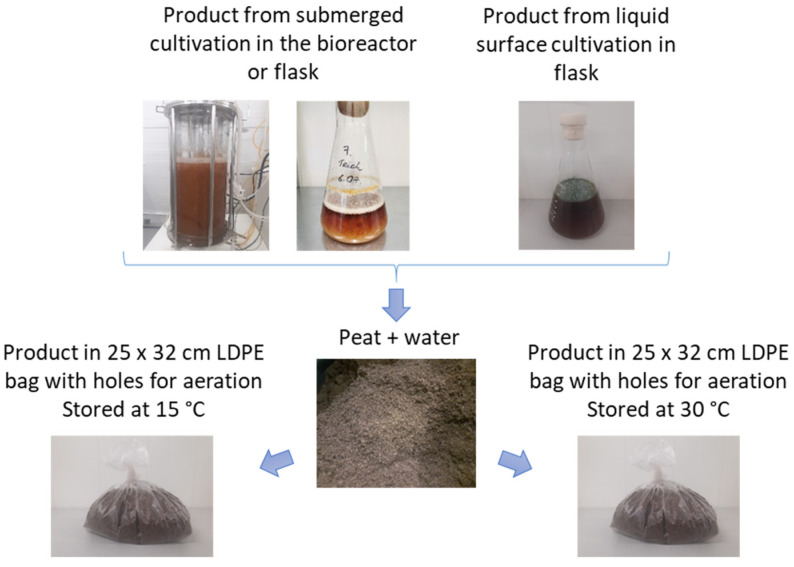
A schematic illustration of the storage experiments.

A schematic illustration of the storage experiments is shown in Figure 1. To 1 kg of peat, 150 mL of the *T. asperellum* product obtained in the process of submerged cultivation in bioreactors or flasks, 540 mL of water, and 35 mL of the obtained mass of statically cultured *T. asperellum* (7 days, in malt extract medium, at 25 °C) were added, yielding approximately 1.76 kg of peat product.

From the obtained mass, 700 g was weighed in a 25 × 32 cm LDPE bag, in which holes were punched every 5 cm for aeration. Peat sample bags were stored at 15 ± 3 °C for one year and at 30 °C in an incubator (Heraeus Instruments D-6450 Hanau, Germany) for 70 days. From each peat sample that was kept at 30 °C (in three replicates), on days 0, 10, 20, 30, 50, and 70, after the application of *T. asperellum* biomass to the peat, measurements were taken to determine changes in the number of CFU over time. From each peat sample that was kept at 15 °C (in three replicates), on days 0 and 365, analysis for CFU was performed. Peat samples kept for 365 days were stored at 30 °C for 10 days, after which the changes in the number of CFU over the incubation time were analyzed.

### 2.5. Analytical Measurements

To detect *T. asperellum* dry weight from cultivation in the bioreactor and flasks, culture consisting of medium and hyphae with chlamydospores was centrifuged for 10 min at 3000 rpm to separate the supernatant. Moisture content was determined by heating the biomass in an AGS 120/T250 moisture analyzer (Axis, Gdańsk, Poland) at 85 °C for 25 min. To detect dry weight from the biomass on the Petri dish, a method from Senkovs et al. (2022) [36] was used. In each experiment, the highest biomass reached was taken as 100% dry weight, and percentages were adjusted respectively for all biomass from other media.

To evaluate the effect of media and Tween 80 on the capacity of *T. asperellum* to form spores on the biomass surface, biomass was placed on Petri dishes (in three replicates) and stored for five days. The biomass was obtained by filtering the culture liquid through a polyamide mesh with a pore size of 0.7 mm × 0.7 mm, obtaining biomass with 86% humidity. To determine the quantity of spores and hyphal fragments, the biomass was mixed and then dried in a one cm-thick layer for three days on a metal sieve with a pore size of 0.7 mm × 0.7 mm at 28 °C. After the biomass dried completely, it was ground until it became a powder in a coffee grinder. The quality of powder was determined by the number of CFU/g.

To determine the number of colony-forming units (CFU/g) of *T. asperellum,* inoculations on the universal medium for fungal growth (Malt extract agar) at different dilutions were performed.

### 2.6. Statistical Data Processing and Analysis

All experiments and measurements were performed in three replicates. To determine whether there was a statistically significant difference between the measured parameters, the computer program RStudio (version 1.4.1103, R Foundation for Statistical Computing, Austria) was used, which calculated the values of standard deviations and compared their agreement between different samples.

## 3. Results

This study investigated *T. asperellum* cultured in eight different media, stored with or without incorporation in a carrier material, and the formation of conidia was monitored in these samples. In addition, the effect of the culture medium on the ability of the biomass to form conidia without embedding in the carrier was studied. The ways in which the cultivation environment and biomass yield affected the viability of the culture when it was embedded in peat and stored at 15 °C and 30 °C were also investigated.

### 3.1. Trichoderma asperellum Biomass Cultivation in Shake Flasks and Storage Tests

M1, M2, M3, and M4 media (with or without 1% Tween) were used to determine the best cultivation environment for *T. asperellum* biomass growth and its storage in peat. An increase in biomass was observed in the MT1, MT2, and MT4 media, and a decrease was observed in the MT3 medium (Table 1). It could be observed that the cultivation medium is especially important regarding the formation of conidia.

**Figure 2:**
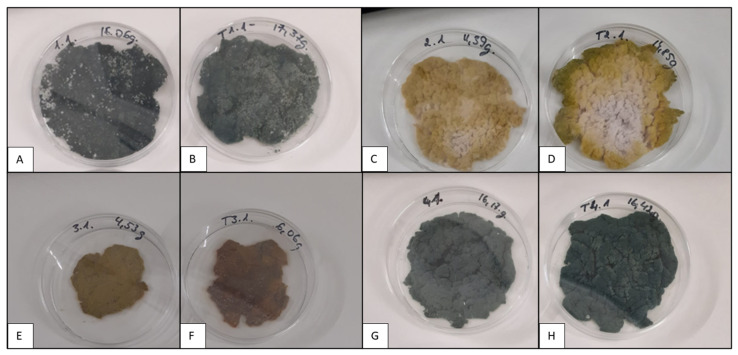
The morphological features of *T. asperellum* biomass (cultivated in flasks) depending on the composition of the culture medium after five days. Individual picture designation regarding the medium used: (**A**)—medium M1, (**B**)—medium MT1, (**C**)—medium M2, (**D**)—medium MT2, (**E**)—medium M3, (**F**)—medium MT3, (**G**)—medium M4, (**H**)—medium MT4. M1—malt extract medium, M2—yeast extract medium, M3—salt medium, M4—molasses medium, MT1—malt extract medium with 1% Tween 80, M2—yeast extract medium with 1% Tween 80, M3—salt medium with 1% Tween 80, and M4—molasses medium with 1% Tween 80.

In determining the number of CFU in the spore powder obtained from the biomass that displayed conidia formation, M1/MT1 and M4/MT4 yielded 10^8^ CFU/g, whereas M2/MT2 and M3/MT3 yielded only 10^4^ CFU/g. A positive effect from Tween 80 on conidia development could be visually observed in the biomass that was cultivated in the MT2 medium (Figure 2). However, analysis after drying showed that the number of CFU in MT2 was not significantly higher than that in M2 (*p* = 0.8914).

**Figure 3:**
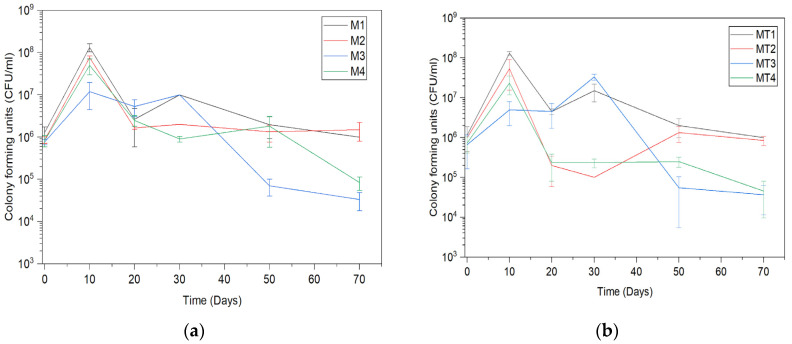
CFU counts in peat during its storage at 30 °C in (**a**) medium without 1% Tween 80, (**b**) medium with 1% Tween 80. Medum designations: MT1—malt extract medium with 1% Tween 80, M2—yeast extract medium with 1% Tween 80, M3—salt medium with 1% Tween 80, and M4—molasses medium with 1% Tween 80.

After the incorporation of biomass from the samples obtained from the M1 and M2 cultivations into peat and storage for 70 days at 30 °C, no significant changes in the amount of CFU could be observed with the addition of Tween 80. Significant changes in M3/MT3 after incorporation into peat could be observed only on day 30 (*p* = 0.0120), but in M4/MT4, significant differences could be observed already on day 20 (*p* = 0.0100) and 30 (*p* = 0.0040) (Figure 3). When CFU analyses were performed immediately after incorporation in the peat, no significant differences in results between different media were observed. After 10 days of peat storage at 30 °C, all samples showed an increase in the number of CFU. The smallest increase after 10 days was observed in M3. Statistically significant differences were observed between the M1 and M3/M4 (*p* = 0.0005 and 0.0026, respectively) media. Significant differences were also observed using media with Tween 80 added at a concentration of 1%, with the exception of MT2 and MT4 (*p* = 0.0907). The dry weight increase correlated with the increase in CFU (Figure 3). Thus, the highest CFU amount was observed in the M1/MT1 media, displaying a biomass of 90.54 ± 34.94% and 100 ± 13.13%, respectively, and the slightest increase was observed for the M3/MT3 media, for which the biomass was 34.56 ± 18.34% and 24.32 ± 7.34%, respectively. The amount of CFU in M2/MT2 and M4/MT4 was in an intermediate position. On day 30, a significant decrease in the amount of CFU was observed in M2 compared to M3 (*p* = 0.0040), M4 compared to M1 (*p* = 0.0290) and M3 (*p* = 0.0006), MT2 compared to MT1 (*p* = 0.0001) and MT3 (*p* = 0.0007), and MT4 compared to MT1 (*p* = 0.0001) and MT3 (*p* = 0.0001). After 50 days, a significant decrease in the amount of CFU was observed in MT3 compared to MT1 (*p* = 0.0350) and MT2 (*p* = 0.0190) and in M3 compared to M4 (*p* = 0.0230). On day 70, differences were observed between all samples, except between M1 and M2, M3 and M4, MT1 and MT2, and MT3 and MT4. On day 70, CFU of the *T. asperellum* grown on the M1, MT1, M2, and MT2 media returned to their initial levels. In contrast, in the M3, MT3, M4, and MT4 media, the amount of CFU decreased compared to the initial reading (Figure 3).

### 3.2. Trichoderma asperellum Biomass Cultivation in a Bioreactor and Storage Tests

An increase in biomass was observed in all media with 0.2% Tween 80 (Table 2). The increase in dry biomass (*p* = 0.0015) in MT1 was statistically significant. Among the other samples, no significant changes in dry biomass concentration could be observed under the influence of Tween 80.

**Figure 4:**
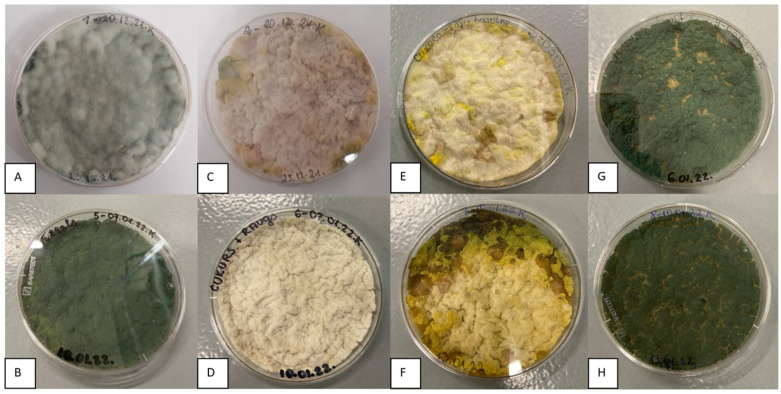
The morphological features of *T. asperellum* biomass (cultivated in bioreactor) depending on the composition of the culture medium after 5 days. The biomass was transferred to a Petri plate prior to adjustment of pH to 4. Individual picture designation regarding the medium used: (**A**)—medium M1, (**B**)—medium MT1, (**C**)—medium M2, (**D**)—medium MT2, (**E**)—medium M3, (**F**)—medium MT3, (**G**)—medium M4, (**H**)—medium MT4. M1—malt extract medium, M2—yeast extract medium, M3—salt medium, M4—molasses medium, MT1—malt extract medium with 1% Tween 80, M2—yeast extract medium with 1% Tween 80, M3—salt medium with 1% Tween 80, and M4—molasses medium with 1% Tween 80.

The biomass grown in bioreactors also sporulated on Petri dishes (Figure 4), similar to the biomass grown in flasks (Figure 2). Green conidia were fully formed only in the biomass cultured on malt and molasses media (M1/MT1 and M4/MT4). The biomass from other experiments retained a white mycelium or yellowish conidial layer.

**Figure 5:**
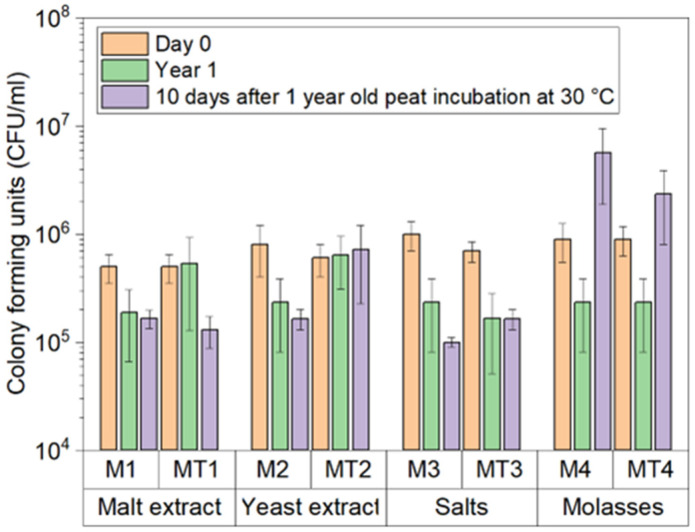
CFU counts in peat after 1 year of storage at 15 °C. After 356 days, peat stored at 15 °C was stored at 30 °C for 10 days, after which CFU analyses were performed. Medium designations: M1—malt extract medium, M2—yeast extract medium, M3—salt medium, M4—molasses medium, MT1—malt extract medium with 1% Tween 80, M2—yeast extract medium with 1% Tween 80, M3—salt medium with 1% Tween 80, and M4—molasses medium with 1% Tween 80.

The pH adjustment to 4 at the end of cultivation in the bioreactor decreased the initial (day 0) CFU amount (Figure 5) compared to the cultivation in shake flasks (Figure 3), for which the pH was not adjusted at the end of the cultivation. Although the decrease in pH affected the initial viability, the long-term viability remained unaffected after that. Although the amount of CFU in the samples incorporated in peat dropped slightly during the 1-year-long storage, the log range remained the same as on day 0. An increase in CFU numbers was observed after 10 days of storage of 1-year-old peat at 30 °C in samples from the M2, M4, and MT4 media (Figure 5). Significant differences could be observed between M1/M3 (*p* = 0.026536), M2/M3 (*p* = 0.047896), and M3/MT3 (*p* = 0.047896).

## 4. Discussion

Many factors affect the viability of *Trichoderma* spp. derived from liquid fermentation, of which the cultivation medium and its modifications (e.g., the addition of a surfactant) are considered to be the most important. In this study, different culture media were used for *Trichoderma asperellum* MSCL 309 biomass production, and it was found that different composition and cultivation modes (a flask and a bioreactor) produced diverse biomass yields at the end of 65 h cultivation processes. For both cultivation modes, malt extract and yeast extract media supplemented with Tween 80 (MT1 and MT2) led to the highest biomass concentrations. Overall, the addition of Tween 80 increased the biomass concentration. In the flask experiments with malt extract medium, this led to a biomass concentration about two times higher. The highest biomass concentrations were achieved in the bioreactor cultivations compared to the cultivations in shake flasks.

*T. asperellum* mycelium is characterized by a white color when cultivated with a minimal medium formula, and under starving conditions, dark green spores are formed. When growing in rich medium, *T. asperellum* displayed a bright yellow or orange color until sporulation, which is in accordance with previous results [38]. However, in the present study, no shift in the green color was observed. If the biomass is stored without being incorporated anywhere, the medium in which *T. asperellum* is grown is essential for a longer shelf life of the product, because a higher amount of CFU (10⁸ vs. 10⁴) was found in biomass that produced dark green conidia. The cultivation medium being minimal or rich did not play such a significant role when the cells were incorporated into peat in this study.

Although the biomass yield was increased by the addition of 0.2% and 1% Tween 80 in all four media compositions except for MT3 in shake flask cultivations, the addition of Tween 80 did not influence the fluctuation in the amount of *T. asperellum* CFU during long-term storage at 15 °C or under accelerated storage test conditions at 30 °C. If the addition of Tween 80 to the cultivation medium had contributed to a significantly higher biomass yield, the number of CFU would also be significantly different, and the number of CFU would be much higher in the samples with Tween 80. To significantly increase the number of CFU in the peat, the amount of biomass must be increased, which can be achieved by adding Tween 80 to the culture medium. Our study showed that the addition of 1% or 0.2% Tween 80 is not enough to significantly increase the amount of CFU. Adding higher concentrations of Tween 80 is an economically unprofitable solution. The survival and activity of *Trichoderma* spp. in soil may be influenced by abiotic factors (i.e., unfavorable pH and temperature [39], drought [40], UV radiation [41], salinity [41], pesticide residues [42]) and biotic factors (i.e., cultivated plant species, the soil microbial community) that can affect the survival and persistence of the fungus in soil [43]. To minimize these risks, the formulation must provide competitive advantages to facilitate survival under adverse environmental conditions. Despite some studies showing peat as an unfavorable medium for cell maintenance [44,45], the findings of this study do not support this view. It was not the ability to form green conidia on plates that determined the increase in the amount of CFU, but the amount of biomass that was incorporated. This indicates that peat is a favorable medium for maintaining and even enhancing the viability of cells obtained from submerged cultivations using any of the cultivation media in this study, which is in accordance with previous reports [46].

The results showed that *T. asperellum* biomass mixed in peat was viable after 1 year. Biomass from the molasses medium even displayed an increased number of CFU after 10 days of incubation at a temperature and humidity suitable for spore development. It can be concluded that after one year of peat-based formulation storage at 15 °C under aerobic conditions, the viability is significantly influenced by the cultivation media in which conidia and mycelium containing chlamydospores are obtained.

As shown by accelerated storage tests at 30 °C, the most suitable media for CFU preservation are M1/MT1 and M2/MT2. The amount of CFU measured directly after mixing the biomass with peat likely reflected the amount of conidiospores only, considering that the initial amount of CFU was the same in all the samples. After the incubation at 30 °C for 10 days, the amount of CFU increased proportionally to the dry weight of the biomass obtained from submerged cultivations. Thus, the cell mass develops only after storage in optimal conditions and the incorporated biomass cells start to grow, increasing the amount of CFU. This study did not distinguish between the two spore types, namely, conidiospores and chlamydospores, since storage stability was the main goal of this research. Thus, to increase the number of CFU in the ready product, it can be recommended to incubate the peat-based preparations at 30 °C for 10 days, followed by storage at 15 °C over an extended period of time to maintain a high CFU count.

By adding substances that reduce *T. asperellum* metabolic activity to the biomass, the shelf life of the biomass can be extended, thus maintaining the competitiveness of the microorganisms against plant pathogens. Decreasing the biomass pH can suppress the metabolic rate, thereby improving microbial survival [14,36,47]. This approach, decreasing the pH to 4 at the end of cultivation in a bioreactor, was successfully applied in the present study. Although the pH could not be decreased in the experiments using shake flasks due to sterility hazards, thus rendering the obtained results not directly comparable, the approach was efficient in the large-scale cultivations. Testing the efficacy against plant pathogens displayed by *T. asperellum* cells incorporated in peat and subjected to storage remains a task for further studies.

## 5. Conclusions

A method to achieve long-term viability of *Trichoderma asperellum* incorporated in peat was presented, in which the quality of the product (in terms of CFU) dropped within only one log over one year. However, additional research is needed to investigate the efficacy of such preparation against plant pathogens.

## Figures and Tables

**Table 1:** *Trichoderma asperellum* dry weight (%) from the flask experiment. One hundred percent—highest biomass reached in experiment. The percentage of other biomasses was adjusted respectively to the highest value. Medium designations: M1—malt extract medium, M2—yeast extract medium, M3—salt medium, M4—molasses medium, MT1—malt extract medium with 1% Tween 80, M2—yeast extract medium with 1% Tween 80, M3—salt medium with 1% Tween 80, and M4—molasses medium with 1% Tween 80.

Medium	Dry Weight, %
M1 (MT1)	91 ± 35 (100 ± 13)
M2 (MT2)	50 ± 18 (77 ± 10)
M3 (MT3)	35 ± 18 (24 ± 7)
M4 (MT4)	56 ± 15 (78 ± 7)

**Table 2:** *Trichoderma asperellum* dry weight (%) from the bioreactor experiment. One hundred percent—highest biomass reached in experiment. The percentage of other biomasses was adjusted respectively to the highest value.

Medium	Dry Weight, %
M1 (MT1)	51 ± 4 (100 ± 10)
M2 (MT2)	70 ± 3 (76 ± 3)
M3 (MT3)	43 ± 5 (55 ± 5)
M4 (MT4)	30 ± 5 (42 ± 6)

## Data Availability

The data presented in this study are available on request from the corresponding author. The data are not publicly available due to company ownership.
